# Rare novel ***LPL*** mutations are associated with neonatal onset lipoprotein lipase (LPL) deficiency in two cases

**DOI:** 10.1186/s12887-021-02875-x

**Published:** 2021-09-20

**Authors:** Yun Qin Wu, Yue Yuan Hu, Gui Nan Li

**Affiliations:** Department of Neonatology, Hunan Children‘s Hospital, No.86 Ziyuan Road, Changsha, 410007 China

**Keywords:** Lipoprotein lipase deficiency, Hypertriglyceridemia, Neonatal period, Recurrent infection

## Abstract

**Background:**

Lipoprotein lipase (LPL) deficiency is a monogenic lipid metabolism disorder biochemically characterized by hypertriglyceridemia (HTG) inherited in an autosomal recessive manner. Neonatal onset LPL deficiency is rare. The purpose of this study was to clarify the clinical features of neonatal LPL deficiency and to analyze the genetic characteristics of *LPL* gene.

**Methods:**

In order to reach a definite molecular diagnose, metabolic diseases-related genes were sequenced through gene capture and next generation sequencing. Meanwhile, the clinical characteristics and follow-up results of the two newborns were collected and analyzed.

**Results:**

Three different mutations in the *LPL* gene were identified in the two newborns including a novel compound heterozygous mutation (c.347G > C and c.472 T > G) and a reported homozygous mutation (c.836 T > G) was identified. Interestingly, both the two neonatal onset LPL deficiency patients presented with suffered recurrent infection in the hyperlipidemia stage, which was not usually found in childhood or adulthood onset LPL deficiency patients.

**Conclusion:**

The two novel mutaitons, c.347G > C and c.472 T > G, identified in this study were novel, which expanded the *LPL* gene mutation spectrum. In addition, suffered recurrent infection in the hyperlipidemia stage implied a certain correlation between immune deficiency and lipid metabolism abnormality. This observation further supplemented and expanded the clinical manifestations of LPL deficiency.

**Supplementary Information:**

The online version contains supplementary material available at 10.1186/s12887-021-02875-x.

## Background

Lipoprotein lipase (LPL) deficiency is a lipoprotein metabolism disorder inherited in an autosomal recessive manner caused by dysfunction of the *LPL* gene. This rare disease is often diagnosed in childhood and has an incidence of about 1:1,000,000 [1]. Patients with LPL usually have elevated plasma triglycerides (TG), abdominal pain, recurrent acute pancreatitis, exanthematous xanthoma, and hepatosplenomegaly [2]. Diagnosis of neonatal-onset LPL deficiency is extremely rare [3]. It is often diagnosed as chylaemia based on the routine blood tests or occasional ivory plasma found in laboratory tests. Complex heterozygous or homozygous mutations in the *LPL* gene can be detected in most patients through gene sequencing [4].

The *LPL* gene is located in the 8p22 band and contains 10 exons and 9 introns spanning about 30 kb [5]. The protein encoded by *LPL* gene is the rate-limiting enzyme that is degraded into glycerol and free fatty acids (FFA) and is mainly produced and secreted by cardiac muscle, skeletal muscle, and adipose tissue [6]. In organ parenchymal cells, it is secreted and transported to the luminal surface of vascular endothelial cells, where it is embedded in the inner wall of blood vessels through ion reaction with heparan sulfate proteoglycan or glycosylated phosphoinositide [7]. In the capillary endothelium, it mainly acts on TG-rich lipoproteins in the plasma, such as chylomicrons (CM), low-density lipoprotein (LDL), very-low-density lipoprotein (VLDL), etc. Moreover, LPL can exert its lipolytic activity only when combined with apolipoprotein II [8]. Its deficiency or activity decrease can lead to the degradation of plasma CM and VLDL, which in turn leads to an increase in plasma TG and VLDL. Clinical symptoms indicate that the plasma LPL activity of patients with type I hyperlipidemia is not equivalent to 1/10 of the normal level, which may also affect the metabolism of liver cholesterol and thereby further influence the cholesterol and bile acid content in bile. In this study, we analyzed and summarized the clinical manifestations and molecular genetic characteristics of two neonatal patients who were suspected of having LPL deficiency.

## Methods

### Clinical characteristics and follow-up results of the two patients

#### Clinical characteristics

The proband 1, G1P1 male, was admitted to the neonatology department of our hospital at 11 days after birth. He had yellow skin for 10 days and abnormal blood lipids for 4 days. The proband 1, was delivered at 39 weeks of gestation, weighing 3.32 kg. There was no history of perinatal hypoxia and breastfeeding. Yellow skin occurred on the second day after birth. Laboratory results showed that total cholesterol (TC) was 6.81 mmol/L (reference value: 3.00–5.20 mmol/L) and the TG was 12.22 mmol/L (reference value: 0.40–1.70 mmol/L) at 7 day of age. Physical examination showed the child’s weight was 3.7 kg, the length was 50 cm, head circumference was 36 cm. were in normal range; heart and lungs were normal; the liver was 2 cm below the ribs, the spleen was not palpated, and there were no abnormalities in the nervous system. The blood was ivory white to pink after standing. After admission, the blood lipid test results of the proband were significantly abnormal, which are summarized in Table 1. The father was 30 years old, and the mother was 28 years old. Both non-consanguineous parents showed normal behavior and normal blood lipids. The patient’s two-year-old younger brother also presents with normal blood lipids.

**Table 1:** Biochemical test results of the two patients

Biochemical test results				
Case 1				
Time	TC(mmol/L)	TG(mmol/L)	HDL-C(mmol/L)	LDL-C(mmol/L)
Reference	2.59 ~ 5.60	0.49 ~ 2.30	0.82 ~ 2.04	2.50 ~ 3.50
11D	9.99	14.28	–	–
15D	6.18	16.82	–	–
19D	3.57	12.92	–	–
23D	2.93	10.42	–	–
3M25D	2.06	6.97	0.24	1.38
5 M	2.98	6.31	0.49	2.11
9M22D	3.39	10.78	0.57	1.73
10M27D	3.41	11.29^I^	0.51	1.62
1Y3M	4.75	9.97	0.66	1.92
2Y8M	9.28	25.24^I^	0.91	2.18
Case 2				
28D	18.4	140.20	0.22	5.7
31D	1.22	4.02	0.13	0.02
1M17D	2.57	16.97	0.28	0.84
4M11D	4.36	25.09^I^	0.13	1.43
7M12D	3.55	28.05^I^	0.19	1.14
18M5D	3.04	12.57	0.43	1.25
18M14D	–	5.59	–	–

TC indicates: Total cholesterol, TG indicates: Triglyceride, HDL-C indicates: High density liptein cholesterol, LDL-C indicates: Low density liptein cholesterol, Y indicates: Year, M indicates Month, D indicates Day, I indicates: Infection

The proband 2, G3P2 female, was admitted to the neonatology department of our hospital at 28 days after birth due to nasal congestion and cough for 3 days, and abnormal blood lipid for 1 day. The proband 2, was delivered at 37 + 4 weeks of gestation. There was no history of perinatal hypoxia and breastfeeding. She had jaundice (4th day after birth) that the peak serum bilirubin level was 23.5 mg/dl on the 6th day and subsided on the 9th day. Biochemical analyses showed abnormal level of blood lipids. TC, 18.4 mmol/L; TG, 140.20 mmol/L; high density lipoprotein cholesterol, 0.22 mmol/L; LDL, 5.7 mmol/L. Physical examination showed the child’s weight was 3.17 kg, the length was 56 cm, head circumference was 35 cm, were in normal range; heart and lungs were normal; the liver was 1.5 cm below the ribs, the spleen was not palpated, and muscle hypotonia in the extremities was normal and the original reflex could be elicited. After admission, the blood lipid test results of the proband were significantly abnormal, which are summarized in Table 1. The father was 33 years old, and the mother was 29 years old. Both non-consanguineous parents and the patient’s seven-year-old older brother showed normal behavior and normal blood lipids.

#### Follow-up results

The proband 1 was followed up for 3 years. Amino acid-based formulae (AAF) had been taken continuously accompanied by diet control, a low intake of a high-fat diet. He showed normal growth with no jaundice, abdominal pain, and other abnormalities. Blood samples were taken regularly to review the biochemical values of blood lipids, and the blood lipid test results were shown in Table 1. In addition, he experienced several respiratory tract infections when his blood lipids were high.

The proband 2 was followed up for 19 months. And amino acid-based formulae (AAF) had been taken continuously accompanied by diet control. She showed normal growth and development, slightly low. Blood samples were taken regularly to review the biochemical values of blood lipids, and the blood lipid test results were shown in Table 1. Simvastatin was administered at a dose of 10 mg per day at 8 months of age. The blood lipids level is decreasing gradually. At 17 months and 21 days, the triglyceride was a little higher (2.98 mmol/L), other indicators were normal. Simvastatin decreased to 5 mg QD. Two weeks later, TG was 12.57 mmol/l, and the drug dose remained unchanged (simvastatin 5 mg QD). Three weeks later, the blood lipid was reduced to 5.59 mmol/l. Follow-up is maintaining until now. In addition, she experienced several respiratory tract infections when her blood lipids were high too.

### Target next generation sequencing

Genomic DNA was extracted from the peripheral blood by using QIAamp DNA Mini Kit (QIAGEN, Hilden, Germany). DNA was quantified with Nanodrop 2000 (Thermal Fisher Scientific, DE, Germany). 5μg DNA was used for library construction by NEXTflex® Rapid DNA-Seq Kit (BIOO, Texas, USA) according to the manufacturer’s protocol. Target gene were captured by using a custom enrichment kit (IDT, Coralville, Iowa, USA). The enriched libraries were sequenced on an Illumina HiSeq XTen sequencer (Illumina, San Diego, CA, USA) for a depth of 100-200X. Alignment was performed by Short Oligonucleotide Analysis Package (SOAP) aligner software [9]. Snp_indel was called by GATK and annotated by ANNOVAR [10, 11]. Variants were filtered when it met any one of the following criteria: Minor allele frequency > 0.5%, synonymous mutations, Benign or likely benign judged by American College of Medical Genetics (ACMG). Subsequently, suspected pathogenic variants were confirmed by Sanger sequencing.

### Biochemical analysis

Fasting blood samples were collected in the morning, and the 7600 automatic biochemical analyzer was used to determine the content of TC, TG, HDL-C, LDL-C in serum.

## Results

### Analysis of the biochemical test results

The biochemical test results of the two children are shown in Table 1. The serum TC and TG levels of their parents were all normal. Increased blood lipids were found in both cases. In addition, Case 1 had elevated blood lipids accompanied with symptoms of respiratory tract infection.

### *LPL* gene mutation site

**Fig. 1:**
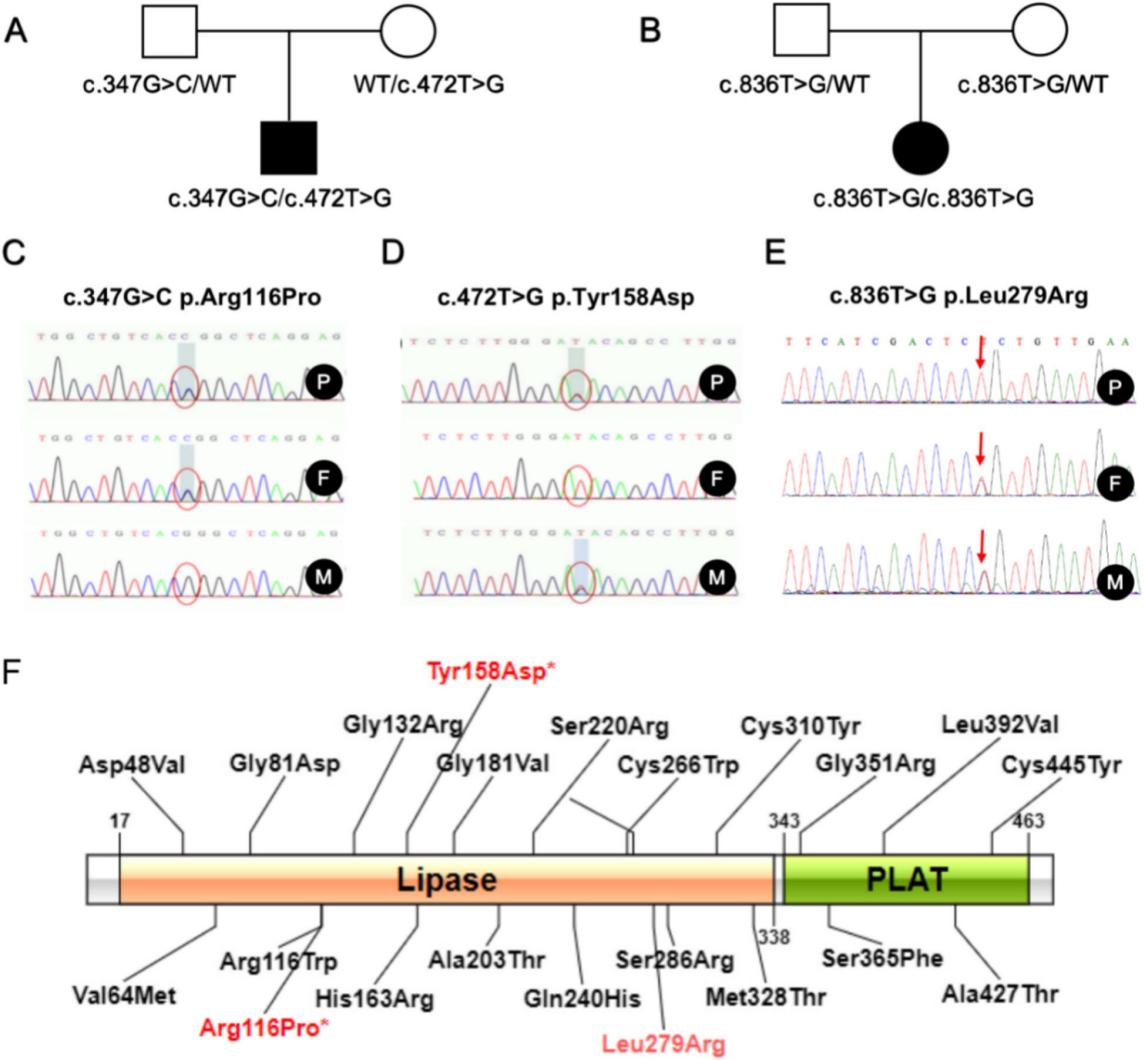
Analysis of *LPL* gene mutation sites in two cases. **A** A pedigree of case 1; **B** Pedigrees of case 2. **C** c. 347G > C family verification results. **D** c.472 T > G family verification results. **E** c.836 T > G Family verification results. **F** Schematic diagram of *LPL* gene mutation sites

Rare mutations in the *LPL* gene were detected in both newborns. One of them had a complex heterozygous variant c.347G > C from the father and c.472 T > G from the mother (Fig. 1A, C, D). c.347G > C was located in exon 3, resulting in the mutation of arginine at position 116 to proline.; c.472 T > G was located in exon 4, causing tyrosine at position 158 muted to aspartic acid (Fig. 1F). The other child, whose parents were both heterozygous carriers (Fig. 1B, E), carried a c.836 T > G homozygous variant located on exon 6, which led to the mutation of 279 from leucine to arginine (Fig. 1F).

### Pathogenicity analysis of *LPL* gene variant sites

**Fig. 2:**
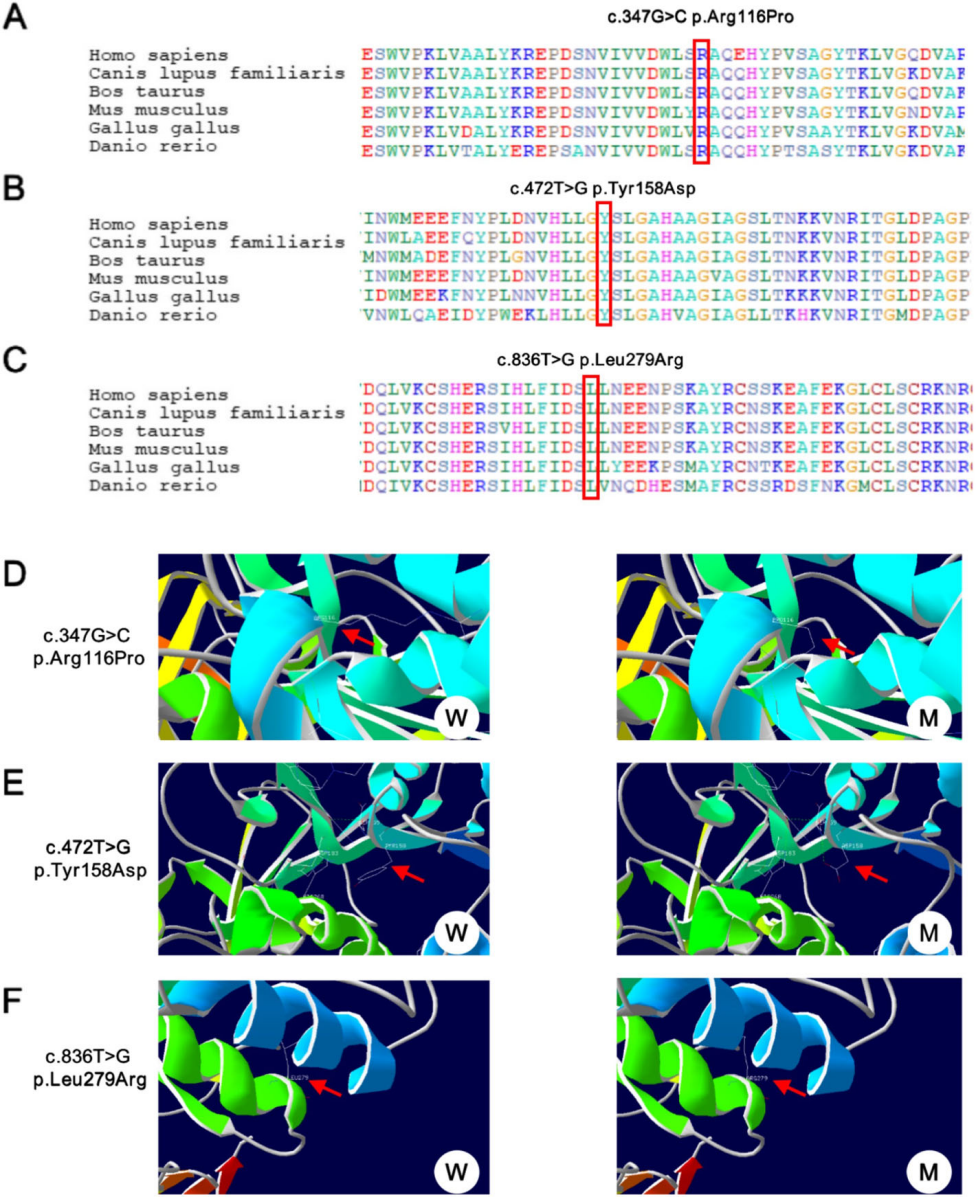
Conservation analysis and tertiary structure prediction of *LPL* gene mutation sites. **A**,**B**,**C** Conservation analysis of c. 347G > C, c.472 T > G and c.836 T > G (**D**,**E**,**F**) tertiary structure prediction of LPL gene c. 347G > C, c.472 T > G and c.836 T > G mutation

The c.347G > C, c.472 T > G, and c.836 T > G mutations affected amino acids the 116, 158, and 279 of the *LPL* gene, respectively, which were highly conserved by conservative analysis of multiple species (Fig. 2A, B, C). Moreover, they were all predicted to be harmful by the software SIFT, PolyPhen, and Mutationtaster. c.347G > C and c.472 T > G have not been previously reported. They were also considered to be detrimental as predicted by SIFT, PolyPhen, and Mutationtaster.

Using SWISS-MODEL to predict the tertiary structure, it was suggested that the mutation of p. Arg116Pro may cause changes in the side chain (Fig. 2D). p.Tyr158 is close to the active catalytic center of LPL; the mutation can influence the formation of a hydrogen bond between Tyr158 and Ser159 (active catalytic site)(Fig. 2E). Though the c.836 T > G mutation site has been reported, it has not been found in multiple population databases and is predicted to be harmful by SIFT, PolyPhen, and Mutationtaster. The prediction of the tertiary structure by SWISS-MODEL indicated that p.Leu279Arg affects its side-chain structure(Fig. 2F). In addition, this site is adjacent to the second disulfide bridge of the *LPL* gene, which may interfere with the formation of disulfide bridges, and thus further affect the correct folding of LPL protein, resulting in abnormal degradation of LPL in cells.

## Discussion

Lipoprotein lipase deficiency is a single-gene rare autosomal recessive disorder of lipid metabolism with HTG due to *LPL* gene dysfunction [12]. *LPL* gene encoded the main lipolytic enzyme which is the rate-limiting step in the removal of triglyceride from the blood stream. LPL deficiency is a major cause of HTG which account for most patients and mostly restricted to patients with type 1 HTG [13]. Beside *LPL* gene, other regulated genes also can cause HTG through affecting the activity or stability of LPL, such as *GPIHBP1*, *LMF1*, *APOC2* and *APOA5* [14]. For example, some HTG patients were caused by the mutations in *APOC2* gene, which plays a critical role in TRL metabolism by acting as a cofactor of lipoprotein lipase (LPL) [15]. The clinical manifestations include abdominal pain, skin xanthomas, or hepatosplenomegaly [2]. In the majority of patients, it occurs before the age of 10, while LPL deficiency in neonates is very rare [3, 10]. Only 25% of neonates have symptoms during the first year of life. So far, only one LPL case with immunodeficiency has been reported in the neonatal period [3]. In this study, we reported two neonatal LPL cases with yellowish skin and dyslipidemia. Different from children or adult patients, both the two probands had repeated infections, which further suggested that immune deficiencies may be found in neonatal patients, thus expanding the clinical manifestations of LPL deficiency in the neonatal period. Through the follow-up period, we found that the increase in blood lipids was more obvious during infection, suggesting a correlation between infection and blood lipid elevation. This higher risk of infection may be due to the immune deficiency attributed to abnormal functions of monocytes, lymphocytes, antigen-presenting cells, and centrifugal cells, which were caused by hyperlipidemia [16–20]. APOE knockout mice were also susceptible to a variety of pathogens, further verifying that hyperlipidemia could affect immune function through a series of signaling pathways [21]. A study conducted in mice showed that cholesterol derivatives could inhibit the inflammatory response mediated by interleukin-1β and that hyperlipidemia had a role in immune function [22]. However, the specific molecular mechanism of immune deficiency resulted from hyperlipidemia is still unclear and needs further investigation.

Both probands and their parents underwent high-throughput and Sanger sequencing. The *LPL* gene Arg116Pro and Tyr158Asp complex heterozygous variants, and Leu279Arg homozygous mutations were detected in the two families, respectively. At present, the secondary structure of LPL is not clear. The LPL molecule is composed of two structural regions: the N-terminal region and the C-terminal region [23]. As an important functional area of LPL, the N-terminal region consists of amino acids 1 to 315, which form a nearly spherical shape structure dominated by β-sheets. The LPL catalytic activity center is composed of Ser159, Asp183, and His268 is located in this area. Studies have shown that the LPL activity was significantly reduced or even disappeared if neutral amino acids substituted amino acids near the active center. Besides, Asn43 is an important glycosylation site in the N-terminal region, which has a role in maintaining the normal three-dimensional structure of LPL and is of great significance to the activity of normally secreted LPL. The C-terminal region is a folded column connected to the inner of the spherical N-terminal region; yet, its exact function remains unknown. It is believed that this is related to the binding of enzymes to the substrate. Through the C-terminal (279–282 and 292–304), the heparin-binding domain is formed to mediate the binding of LPL to its substrate lipoprotein [24]. In addition, the C-terminal region is necessary for the transfer of newly synthesized LPL from the rough endoplasmic reticulum to the Golgi apparatus and then to the extracellular secretion. Abnormal C-terminal structure caused by C-terminal missense mutations, or C-terminal truncation due to C-terminal truncation mutations will lead to the retention of LPL in the rough endoplasmic reticulum and blocked extracellular secretion, thereby affecting the content of extracellular LPL.

The Arg116 site is a highly conserved amino acid site in LPL. Rabacchi et al performed *LPL* genetic testing on 149 patients with severe HTG (TG > 10 mmol/L) and 106 patients with moderate HTG (TG > 4.5 and < 10 mmol/L) [25]. Arg116Gln heterozygous variant was found in a 38-year-old female patient accompanied by severe HTG (TG = 13 mmol/L), whose 75-year-old father was also carrying the same type of variant (the father TG was.49 mmol/L). Furthermore, Evans et al conducted *LPL* genetic testing on 434 patients with dyslipidemia; Arg116Trp heterozygous variation was found in a 48-year-old female patient (with TG 7.84 mmol/l, TC6.68 mmol/l, HDL0.77 mmol/l, NonHDL5.91 mmol/l) [26]. This variant site was not seen in multiple normal population databases and proved to be harmful according to the software of SIFT, PolyPhen, and Mutationtaster. The tertiary structure prediction showed that this mutation might cause changes in the side chain and was suggested as a pathogenic variant.

Tyr158 is a highly conserved amino acid site of LPL that is adjacent to the active catalytic center of LPL (Ser159, Asp183, and His268). Several previous studies reported that multiple variant sites around this region affect the catalytic activity of LPL, such as Leu155Pro, Gly161Glu, and His163Arg [27–29]. SIFT, PolyPhen, Mutationtaster and other software suggested that this mutation is harmful; the tertiary structure prediction results suggested that the mutation of p.Tyr158Asp influenced the formation of hydrogen bonds between Tyr158 and Ser159 (catalytically active site), which might decrease the catalytic activity of LPL. Ma et al showed that the substitution of positively charged arginine for non-polar Leu at position 279 might affect the correct folding of LPL and lead to loss of catalytic activity *in vitro* [30]. After transfecting Leu279Arg cDNA in COS-1 cells, Chan et al did not detect LPL activity in the cell culture medium and lysate. It was speculated that Leu279Arg could influence the stability of LPL dimers and cause the lack of mutant protein secretion [31]. The heparin-binding site of LPL is located at residues 279–282 and 292–304 in the N-terminal region; Leu279Arg is located in the N-terminal heparin-binding region. This may induce the charge transfer near the heparin-binding site, affect the conformational stability of LPL, and reduce the affinity of LPL and heparin. It is necessary to combine normal LPL with heparin so as to separate it from parenchymal cells. If the process is disturbed, LPL cannot be outside of the cell and exert its lipolytic activity. Qin et al reported a Chinese family with high plasma triglycerides linked to Leu279Arg heterozygous mutation [31]. In this family, both sister and the father were carriers of the Leu279Arg heterozygous mutation. The child had severe high HTG, and the sister showed moderately elevated TG. At the same time, the father was normal in TG level, which was similar to the proband 2 of our report in whose family there were also differences in the TG levels among Leu279Arg heterozygous mutation carriers. This difference may be related to APOE genotyping or environmental factors.

Diet control and drug therapy with statins are the two main treatment options for children with hypertriglyceridemia. In this study, patients were treated with diet and medication, respectively. Both proband 1 and proband 2 received Neocate powder during infant period. Proband 1 was never treated with lipid-lowering drugs. Proband 2 was treated with simvastatin. Compared with the outcomes of two probands, both diet control and drug therapy obtained definite curative effect. Simvastatin can get better curative effect in a short term. Yet, long exposure and uncontrolled dosage may cause rhabdomyolysis, memory impairment, and liver injury, especially in children. With reference to different medications, fenofibrate has been commonly used to treat adults with HTG. Yet, so far, no standard guidelines have been developed for children.

## Conclusion

In summary, this study analyzed the clinical symptoms, signs, and laboratory test results of two neonatal-onset children with LPL deficiency. Through the target area capture and sequencing technology, the molecular genetic etiology of the two cases was identified. All the three mutation sites were located in the catalytic domain and were predicted as damaging, which implied that might lead to a complete or near-complete absence of catalytic activity. Different from previously reported cases, we reported two cases of neonatal onset LPL deficiency with additional clinical manifestations of recurrent infections, which are rare and further expanding the clinical manifestations of newborns with this disease.

## Supplementary Information

**Additional file 1.**

## Data Availability

Not Applicable.
